# High prevalence of subclinical thyroid nodular disease in autopsies from Mexico with low frequency of NIFTP

**DOI:** 10.3389/fonc.2026.1619965

**Published:** 2026-04-15

**Authors:** Javier Ríos-Valencia, Juan de Dios Bolaños-de-la-Torre, Beatriz Sánchez-Hernández, Claudia I. Rivas-Ortiz, Armando Gamboa-Domínguez

**Affiliations:** Department of Pathology, Instituto Nacional de Ciencias Médicas y Nutrición Salvador Zubirán, Ciudad de México, Mexico

**Keywords:** autopsy, Mexico, NIFTP, noninvasive follicular thyroid neoplasm with papillary-like nuclear features, thyroid nodular disease

## Abstract

**Introduction:**

The prevalence of thyroid nodular disease (TND) varies depending on study type. Nevertheless, a study including autopsies of patients from all over the country, covering almost three decades, is required to deepen our understanding of benign, borderline, and malignant lesions after the World Health Organization 2022 update. This study aimed to identify the prevalence of follicular TND (FTND), borderline, and malignant nodules in autopsies from a third-level hospital in Mexico City, as well as to determine the association between salt iodination and thyroid nodule genesis.

**Methods:**

Autopsies performed between 1992 and 2019 were considered if archived thyroid remnants and clinical data were available. Cases with known premortem thyroid pathology were excluded. Nodules were considered diagnosed if gland morphology was grossly distorted.

**Results:**

The study included 487 autopsies, of which 276/59.2% were women. The mean age was 46.6 years. Of 487 glands, 266 (55%) had TND. Nodular glands had a higher weight than normal glands (17.8 vs. 16.1 g), with a 1.9:1 female-to-male ratio. No increase in the prevalence of nodules was observed after 2004 when salt iodination was regulated (0.40 vs. 0.39; p = 0.969). The median age of patients with FTND was 49 years for men and 51 years for women. Papillary thyroid carcinoma was observed in 44/9.4% glands, and the recently characterized non-invasive follicular thyroid neoplasm with papillary-like nuclear features (NIFTP)/an indeterminate lesion was only identified in seven (1.5%) cases.

**Conclusions:**

TND was not associated with salt iodine regulation in Mexico, and prospective studies are needed to explain this finding in this country. Understanding the prevalence of subclinical FTND, NIFTP, and carcinomas is relevant and can contribute to reducing the rate of overdiagnosis in thyroid pathology.

## 1 Introduction

The knowledge of thyroid nodular disease (TND) prevalence data is valuable when a screening assessment is performed. Its prevalence is related to age, gender, location, study type, and its biases. A cohort study identified a prevalence of 4.2% (N = 218) non-toxic thyroid nodules in the USA, with a 15-year incidence rate of 1.4% (N = 67) non-malignant thyroid nodules (1). A cross-sectional study of 2, 779 persons in Whickham, UK, identified a 6.1% prevalence of palpable thyroid nodules, with its frequency increasing after the age of 45 years (2). An analysis of 477 Swedish middle-aged women living in non-endemic areas of thyroid enlargement showed a goitrous prevalence of 11.3% and a 6.5% prevalence of palpable solitary nodule (3). A more recent and detailed study showed that the prevalence of TND increases with advancing age, demonstrating an 1.6% annual increase in risk for multinodularity (4).

However, the absence of nodules on the physical examination of the thyroid does not always mean that there are no underlying abnormalities. A seminal autopsy study grossing 821 clinically normal glands identified 49.5% nodules (single 12.2% and multiple 37.3%) and demonstrated that thyroid weight is age dependent and that nodularity increases with advancing age, with it being higher in female than male glands (5). Sonographic studies are in line with this figure (35.6% nodules or echo gland abnormalities), and lately, this method has helped to guide thyroid nodule sampling, facilitating the identification of those at risk of malignancy (6). Information on what was previously designated as a hyperplastic nodule and is now called follicular thyroid nodular disease (FTND), as well as on thyroid nodules of any type in autopsy studies in Latin America, is scarce and lacks the use of current nomenclature.

An autopsy study in Western Mexico identified 123 thyroid nodules in 300 necropsies (41%) with a mean age of 37 years (7) and a higher frequency in male patients, with 71% of them found in patients younger than 45 years. However, an epidemiologic study palpating the gland of either gender identified 34 (1.4%) nodules in 2, 401 persons, mainly in young women from Valle de Mexico (8). In our setting and in the surgical pathology arena, benign nodules were found in 732 thyroid surgeries, with an incidence density of 0.05 persons per year for nodule development (9).

Endemic goiter is still recognized in some clusters in the country (10). However, better table salt iodination in 2004 is cited as the cause of its mostly marginal status in recent years (11). Although previous data on FTND phenotype exist (12, 13), no data on non-invasive follicular thyroid neoplasm with papillary-like nuclear feature (NIFTP) prevalence are available from autopsies. These data could be helpful in the diagnostic approach of fine-needle aspirations in suspicious nodules. A study including patients from all over the country across more than two decades is necessary to deepen our understanding of the prevalence of benign, borderline, or malignant thyroid nodules.

## 2 Materials and methods

Pathology records, electronic records, and physical files from 01/01/1992 to 31/12/2019 were analyzed. Inclusion criteria were a) a complete medical record, b) sufficient remnant thyroid tissue to complete at least 10 tissue blocks for histopathological analysis, c) an autopsy report with thyroid weight and gross photography of the gland, and d) without clinical evidence of thyroid pathology in the records. Cases with known premortem thyroid pathology and cases with insufficient tissue and/or incomplete clinical information were not considered for the study. Glands were considered as presenting nodules when the histology of the gland was distorted, regardless of size, for malignant or borderline nodules, and from 5 mm or over for FTND in line with a sonographic consensus (14). A 5-mm threshold for FTND was used because the authors felt confident in being able to recognize thyroid distortions in a projected photograph. Once all the cases had been analyzed at least twice with a double-head microscope by two pathologists (an intentionally double-blind process), they were collated in an Excel sheet, and the statistical analysis was performed.

The results were analyzed through measures of central tendency and dispersion to describe the population. For descriptive statistics, the arithmetic average is used as a measure of central tendency; the standard deviation is a measure of dispersion. Data are presented as the mean ± SD. Standard two-tailed Student’s t-test and χ^2^ were conducted to determine the significance of the differences between the two groups. A point-biserial correlation was run to determine the relationship between age and TND. Two-sided p < 0.05 was considered statistically significant. All the analyses were performed using the IBM SPSS Statistics software (version 25).

## 3 Results

**Figure 1:**
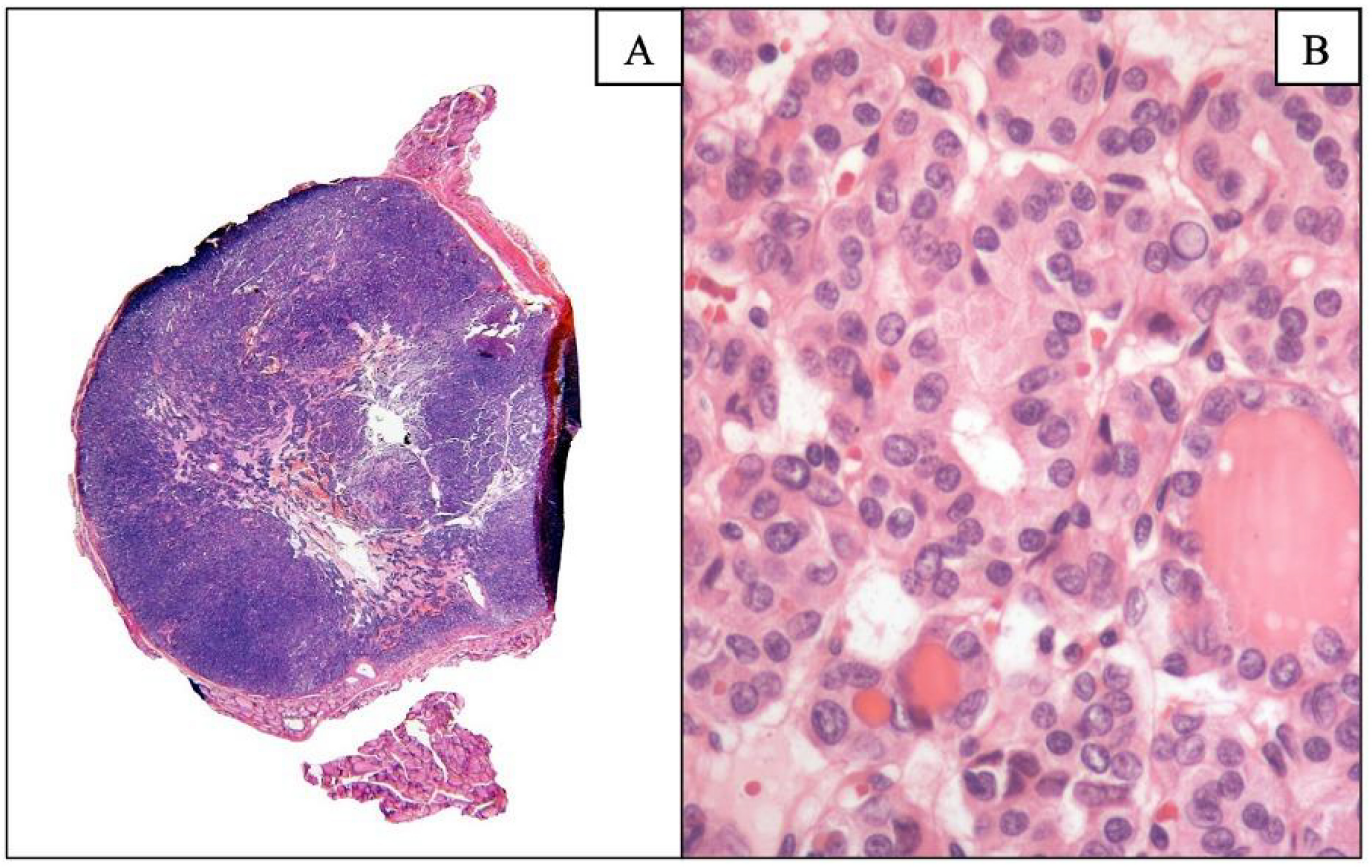
Autopsy trends in a teaching hospital in Mexico City. Panel A shows a low-magnification histological section of a lymphoid tissue with dense purple staining and lighter connective areas, while panel B displays a high-magnification view highlighting round, densely packed nuclei and pink cytoplasm. A decline of postmortem studies was observed from 19% (1992) to 2% (2019) with a change rate of −89% in 27 years*. During COVID-19 pandemic, no autopsies were performed in 2020 for biosafety reasons. In 2021–2022, eight autopsies were performed in 1, 405 hospital deaths (0.5%). *Percentage of rate change was calculated as (end rate − initial rate)/initial rate 100. ** Note. Data from these eight autopsies performed in 2021–2022 were not part of the analysis. Data are shown to illustrate how COVID-19 impacted autopsies in our setting (33).

During the period covered by the study (1992–2019), 913 autopsies were performed with a decline in their performance (**Figure 1**; eight autopsies performed during COVID-19 were graphed but not included) (33). A total of 466 autopsies met the inclusion criteria. The average age of death was 46.6 years (range of 18 to 101 years). Mean thyroid weight was 16.8 g. One or more thyroid nodules and/or NIFTP/malignancies were identified in 55% autopsies.

### 3.1 Subclinical thyroid nodular disease/benign nodules

**Figure 2:**
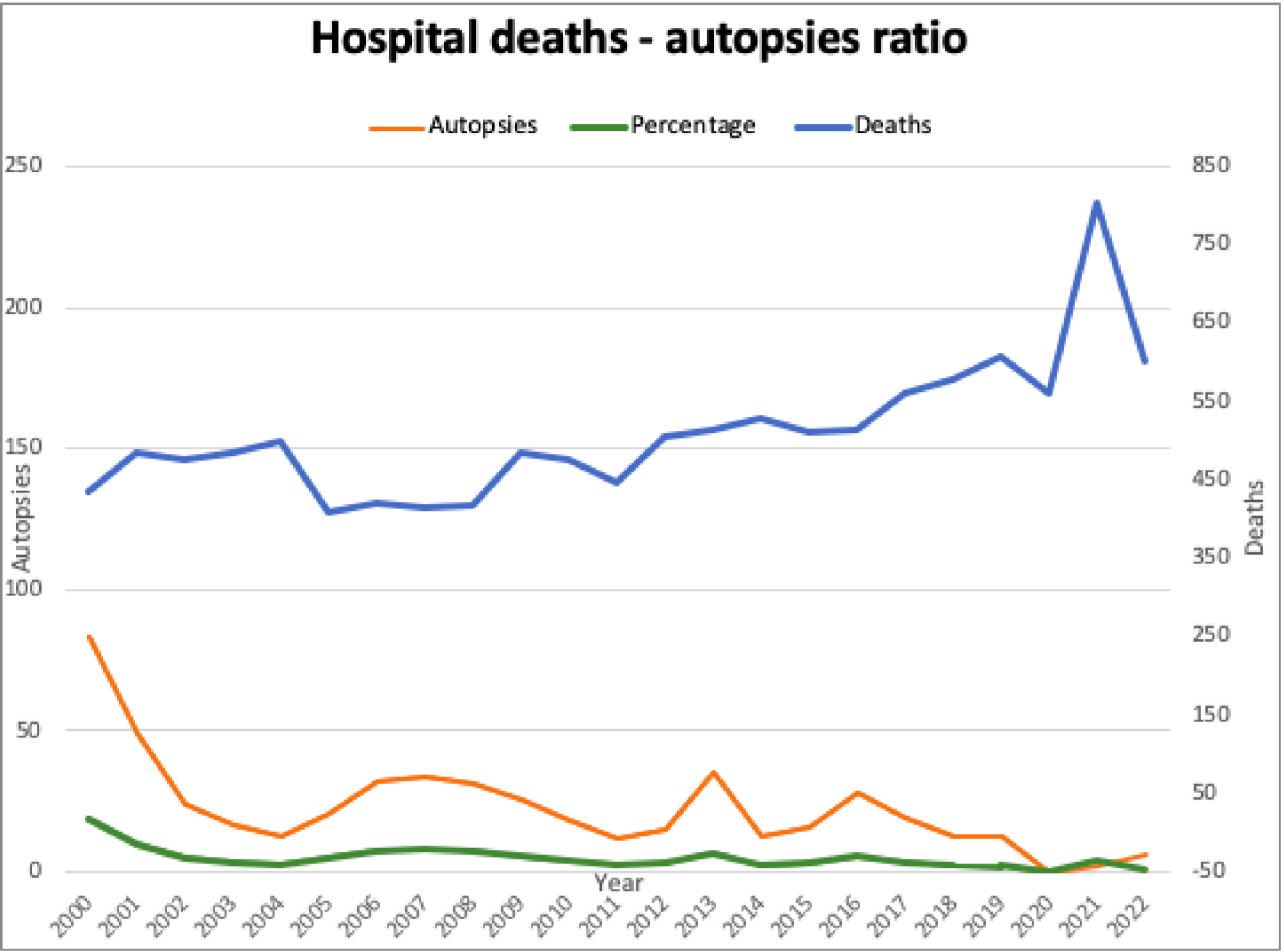
Thyroid nodule genesis and age. An increase in thyroid nodules (one/uTND or multiple/mTND) in autopsies can be observed as the population ages.

Benign thyroid nodules were observed in 216 autopsies (43.9%). Multinodular subclinical thyroid nodular disease (mFTND; more than one 5-mm nodule in the gland) was more prevalent (158/216) than uni-nodular subclinical thyroid disease (u-nFTND) (58/216). There was a positive correlation between the age of death and the presence of nodular disease (**Figure 2**) (rpb = 0.959, p < 0.001). No differences were found in the prevalence of FTND with respect to gender (female 56.8% vs. male 43.2%, p = 0.251). No differences in weight were found between glands without and with nodules (16.1 vs. 17.8 g, p = 0.109) (**Table 1**). No increase in thyroid nodules was observed after 2004 when iodine was regularly added to table salt in Mexico (prevalence of nodules before 0.40 vs. 0.39 after regular salt iodination, p = 0.969).

**Table 1:** Demographic characteristics and histopathological findings of the thyroid in 487 autopsies.

	All autopsies (N = 466)	Autopsies with FTND (N = 216)	Autopsies without TND (N = 250)	P *
	Gender, n (%)^Ç^			0.251
Female	276 (59.2%)	134 (62%)	142 (56.8%)	
Male	190 (40.7%)	82 (37.9%)	108 (43.2%)	
Age of death (mean ± SD)	46.6 ± 18.7	53.4 ± 18.6	42.0 ± 17.3	<0.001
Thyroid weight (g) (mean ± SD)	16.8 ± 9.5	17.8 ± 10.7	16.1 ± 8.5	0.109
Borderline/malignant primary thyroid tumors				
NIFTP n (%)	7 (1.5%)			
PTC, all	44 (9.4%)			
>1 cm	12			
<1 cm	32			
Medullary carcinoma n (%)	1 (0.2%)			

TND, thyroid nodular disease; FTND, follicular TND; NIFTP, non-invasive follicular thyroid neoplasm with papillary-like nuclear features; PTC, papillary thyroid carcinoma.

*Significance testing between thyroids with nodular disease and thyroids without nodular disease.

### 3.2 Borderline/malignant primary thyroid lesions

**Figure 3:**
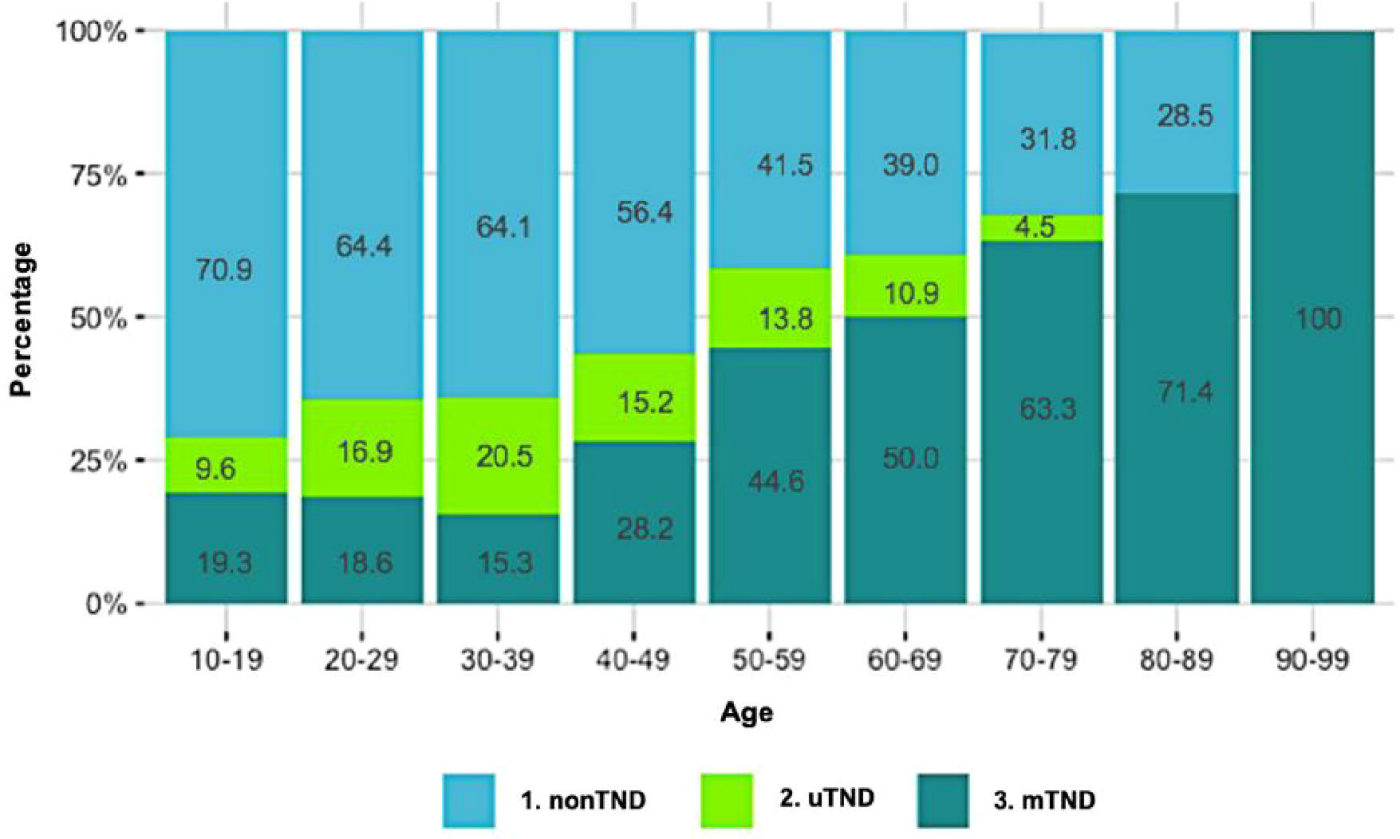
Non-invasive follicular thyroid neoplasm with papillary-like nuclear features (NIFTP). A circumscribed sub-centimetric nodule with follicular growth pattern compressing remnant thyroid tissue can be observed (A: H&E, ×40). Clear chromatin, grooves, and nuclear irregularities were identified in follicular cells (B: H&E, ×400).

Seven cases of NIFTP were found, representing 1.5% of the autopsied population. Three of them also coincide in having papillary thyroid carcinoma (PTC) in the same gland. The mean size of NIFTP was 4.5 mm (**Figure 3**), without prevalence difference between men and women (1.6% vs. 1.8%, p = 0.849) or with the presence of other nodules (2.0% vs. 1%, p = 0.39).

PTC was observed in 44 (9.4%) glands, without gender differences, altogether among thyroids with or without nodules. The mean size of PTC was 5.8 mm. PTCs larger than 10 mm amounted 27.2% (12/44), and 32/44 (72.7%) were smaller. The average size of PTC larger than 10 mm was 14.5 mm. The mean age of death in PTC patients was 47 years. Only one micro-medullary carcinoma was identified in a 57-year-old man whose thyroid gland was otherwise normal. Neither anaplastic nor follicular carcinomas were found.

Infectious thyroiditis with focal necrosis was observed in 15 autopsies from patients with systemic infections by fungus (12), mycobacteria (2), or other bacteria (1). Infiltration by systemic diffuse lymphoma was observed in only one case.

## 4 Discussion

The observed prevalence of TND amounted 55%, and it increases with age. This prevalence is higher than that observed in two previous autopsy studies (49.5% in an American series published 68 years ago and 41% in a more recent study from Western Mexico) (5, 7). The latter is probably related to the older age of the present patients (47 vs. 37 years in the published study), as well as to the smaller threshold of 0.5-mm distorted thyroid structure proposed in the current study. Nodules were more frequently found in women, which is also in contrast with the previous Mexican series that showed 56% (N = 69) nodules in men (7).

Contrary to what was expected (15), no statistically significant differences were observed in the prevalence of nodules before and after 2004, when salt iodization was formally implemented in Mexico. Whether this is due to the short observation period before this regulation (15 years), or to its partial regional implementation in the country, or to other unexplored factors such as iodine concentrations in food, soil, or water, is unknown and outside the scope of this study. These limitations are an inherent part of retrospective studies and should drive prospective efforts to identify whether any causal relationship exists. However, the weight of non-nodular thyroids was 16.1 g, different from the normal thyroid weight reported in other populations (16).

NIFTP were identified in 1.5% (N = 7) of the autopsies, without gender predominance and with an average lesion size of 4.5 mm. Previous publications suggested that this type of lesion would account for 20% of thyroid nodules worldwide when studied using strict diagnostic criteria in lesions larger than 10 mm (12, 13). NIFTP shorter than 1 cm in size was characterized using the revised criteria, and in the re-review of surgical cases, it showed a 6% prevalence in a previous study (17). However, unbiased studies from surgical pathology series have shown a 2.3% prevalence (18), and the epidemiologic approach shows a 10% prevalence of NIFTP among the follicular-patterned thyroid tumors from some regions of the USA (19). Prevalence differences could be explained by variations in morphologic criteria, environmental factors, or ethnicity. However, efforts should be made to explain its 0% to 6.2% prevalence recently observed (20). The same is true in other parts of the world. Studies from Brazil and France have shown frequencies of 15% and 16.9%, respectively (21, 22), whereas in a series of consecutive cases from Argentina, frequencies were 1.2%, and in five Asian countries, they amounted to 2.9% (23, 24). No previous autopsy series were found on this topic, and our findings are in favor of a lower prevalence of NIFTP than expected. This goes along with a population-based study from Denmark showing a 0.7% prevalence of this lesion (25, 26).

In contrast to surgical pathology series, autopsy studies, as demonstrated by this series, do not show a higher prevalence by gender in malignant thyroid tumors. The 9.6% prevalence of malignant subclinical carcinomas identified is higher than the 1.4% and 4% observed in previous reports in Mexico (7, 8), but these are in line with the 4.1% to 11.2% prevalence observed with partial or complete thyroid grossing in a recent autopsy meta-analysis (27). This information is relevant because the availability of high-definition sonographic studies screening sub-centimetric nodules could contribute worldwide to the overdiagnosis and overtreatment of thyroid neoplasms in developed and developing countries (28). In fact, to avoid the risk of overdiagnosis of nodules suspected of malignancy at this setting, our working group initiated multidisciplinary sessions simulating pre-surgical pauses after observing an increase in goiter surgeries (9). We re-reviewed ultrasonography and fine-needle aspiration samples in all listed patients who underwent thyroidectomies, and we noted an improvement in their treatments (29).

The reason why only one subclinical medullary carcinoma was identified, and no case of follicular or anaplastic thyroid carcinoma, lies primarily in the fact that the latter two diseases appear after mutational events of a precursor lesion or progression to an invasive disease (30–32). However, medullary carcinoma is much less frequent than papillary thyroid carcinoma (27). These findings show some strength of the present autopsy series in the unbiased identification of subclinical thyroid nodular disease. At the same time, it is important to acknowledge that the influence of iodine on the presence of thyroid nodules was not recognized with this retrospective study.

In summary, the “normal” thyroid gland weight was 16.1 g, and the prevalence of TND was 55%. The prevalence of NIFTP was 1.5% with a malignancy rate of 9.6% in this autopsy sample of the Mexican population.

## Data Availability

The raw data supporting the conclusions of this article will be made available by the authors, without undue reservation.
